# Supplementary material for: In Vitro Activities and Inoculum Effects of Cefiderocol and Aztreonam-Avibactam against Metallo-β-Lactamase-Producing Enterobacteriaceae
Source: Microbiol Spectr. 2023 May 8;11(3):e00569-23. doi: 10.1128/spectrum.00569-23 (PMC10269523; doi:10.1128/spectrum.00569-23)
Supplement: Supplemental file 1 — Supplemental material. Download spectrum.00569-23-s0001.docx, DOCX file, 0.6 MB [file spectrum.00569-23-s0001.docx]

**Supplementary Table 1**. Microbiological and epidemiological characteristics of the 21 ATM-AVI or cefiderocol or non-susceptible *E. coli* and *E. cloacae complex* isolates

| **Isolate No** | **Species** | **ATM-AVI** | | **CFD** | | **Carbapenemase** | **ST** | **Date of Isolation** | **Specimen Type** | **Ward of CPE isolation** |
| --- | --- | --- | --- | --- | --- | --- | --- | --- | --- | --- |
|  |  | **MIC** | **SIR** | **MIC** | **SIR** |  |  |  |  |  |
| K031 | *E. coli* | 0.125 | S | 8 | I | KPC-2 | 131 | 2020/8/3 | Urine | ICU A |
| M052 | *E. coli* | 1 | S | 16 | R | NDM-5 | 167 | 2018/5/13 | Blood | General ward A |
| M104 | *E. coli* | 8 | I | 8 | I | NDM-1 | 410 | 2020/8/14 | Urine | General ward B |
| M011 | *E. coli* | 4 | S | 8 | I | NDM-1 | 410 | 2016/07/30 | Urine | General ward C |
| M010 | *E. coli* | 4 | S | 16 | R | NDM-1 | 410 | 2016/6/21 | Anal swab | General ward D |
| M055 | *E. coli* | 8 | I | 8 | I | NDM-5 | 617 | 2018/7/14 | Urine | ER |
| M136 | *E. coli* | 1 | S | 16 | R | NDM-5 | 617 | 2020/12/15 | Urine | General ward E |
| **M119*** | ***E. coli*** | **8** | **I** | **4** | **S** | **NDM-5** | **617** | **2020/10/22** | **Urine** | **ER*** |
| **M137*** | ***E. coli*** | **8** | **I** | **2** | **S** | **NDM-5** | **617** | **2020/12/17** | **Urine** | **ER*** |
| M078 | *E. coli* | 16 | R | 4 | S | NDM-5 | 617 | 2019/11/12 | Urine | ER |
| M004 | *E. coli* | 16 | R | 0.125 | S | VIM-1 | 5229 | 2016/2/15 | Anal swab | ICU B |
| M110 | *E. cloacae complex* | 0.25 | S | 16 | R | IMP-8 | 25 | 2020/9/21 | Sputum | General ward F |
| M113 | *E. cloacae complex* | ≤0.06 | S | 8 | I | IMP-8 | 66 | 2020/10/9 | Sputum | General ward G |
| M053 | *E. cloacae complex* | 0.5 | S | 16 | R | NDM-1 | 90 | 2018/5/14 | Sputum | General ward H |
| M103 | *E. cloacae complex* | 0.5 | S | 32 | R | IMP-9 | 90 | 2020/8/8 | Skin pus | General ward I |
| **M089**** | ***E. cloacae complex*** | **0.25** | **S** | **8** | **I** | **NDM-1** | **316** | **2020/3/15** | **Anal swab** | **ICU C**** |
| M155 | *E. cloacae complex* | 0.125 | S | 8 | I | NDM-1 | 316 | 2021/7/1 | Urine | General ward J |
| M085 | *E. cloacae complex* | 0.125 | S | >32 | R | NDM-1 | 316 | 2020/2/10 | Urine | ICU D |
| **M101**** | ***E. cloacae complex*** | **0.25** | **S** | **32** | **R** | **NDM-1** | **316** | **2020/7/25** | **Urine** | **General ward A**** |
| **M124**** | ***E. cloacae complex*** | **0.5** | **S** | **16** | **R** | **NDM-1** | **316** | **2020/11/9** | **Blood** | **General ward G**** |
| M125 | *E. cloacae complex* | ≤0.06 | S | 8 | I | NDM-1 | 1385 | 2020/11/11 | Sputum | General ward K |

**Abbreviations**: ATM-AVI, aztreonam-avibactam; CFD, cefiderocol; CPE, carbapanemase-producing *Enterobacteriaceae*; ER, emergency department; ICU, intensive care unit; MIC, minimum inhibitory concentration; SIR, susceptible, intermediate, or resistant; ST, sequence type

*Before presenting to the ER, the patient carrying M119 was admitted to ward L from 2020/07/07 to 2020/10/12, and the patient carrying M137 was admitted to ward M ward from 2020/11/22 to 2020/12/10. Wards L and M were two adjacent general wards on the same floor.

**The patient carrying M089 was admitted to ward A from 2020/01/18 to 2020/02/20 before being transferred to ICU, the patient carrying M101 was admitted to ward A from 2020/06/16 to 2020/07/28, and the patient carrying M124 was admitted to ward G from 2020/10/18 to 2020/11/23. Wards A and G were two adjacent wards on the same floor. They are the wards for patients with hematologic malignancies.


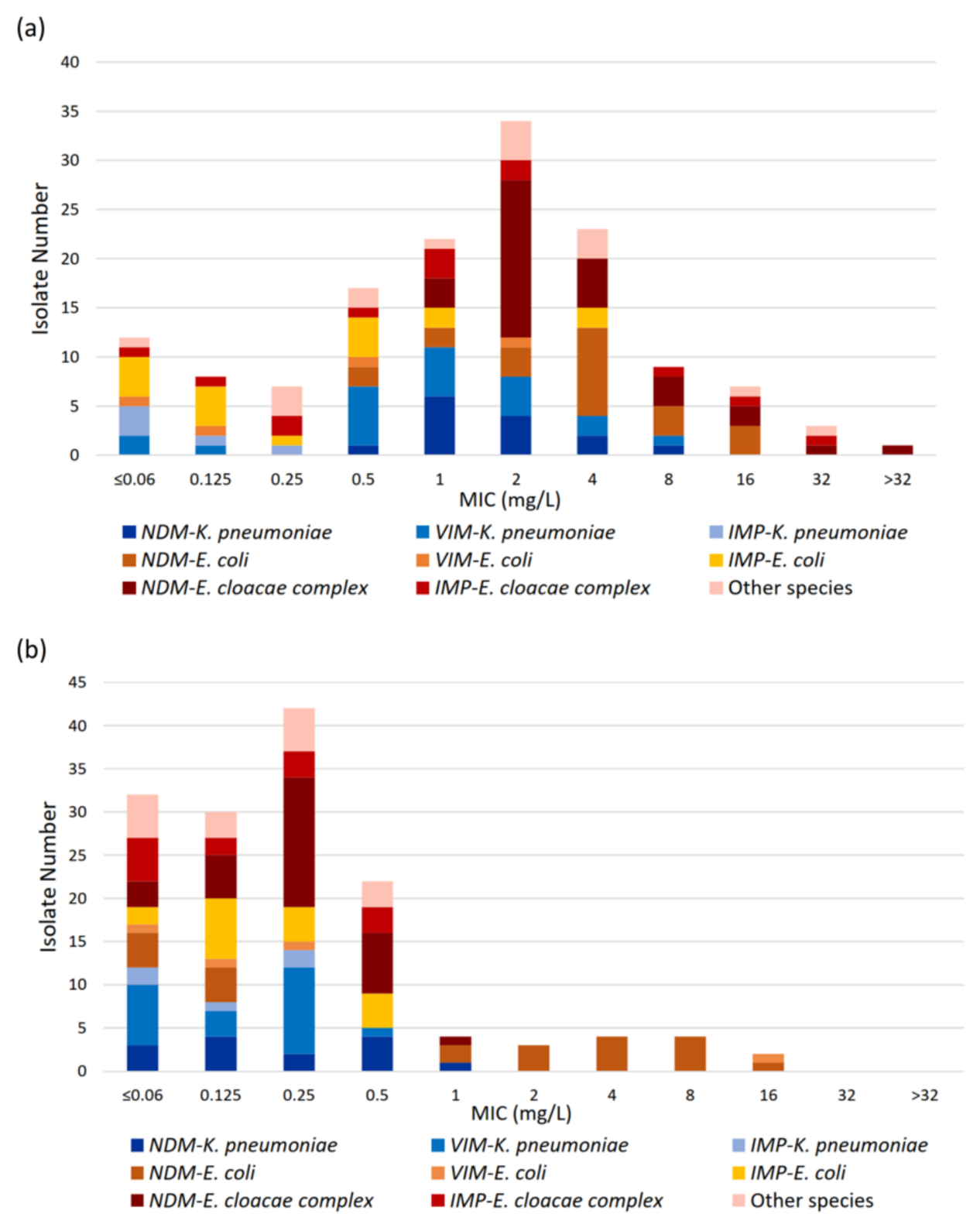
**Supplementary Figure 1**. Minimum inhibitory concentration (MIC) distribution of (A) ceﬁderocol and (B) aztreonam-avibactam among different species of metallo-β-lactamase-producing *Enterobacteriaceae.* Avibactam was tested at a ﬁxed concentration of 4 mg/L.
